# Synthesis Characterization and Photocatalytic Studies of Cobalt Ferrite-Silica-Titania Nanocomposites

**DOI:** 10.3390/nano4020331

**Published:** 2014-04-23

**Authors:** David Greene, Raquel Serrano-Garcia, Joseph Govan, Yurii K. Gun’ko

**Affiliations:** 1 School of Chemistry and CRANN, Trinity College Dublin, Dublin 2, Ireland; E-Mails: greenedj@tcd.ie (D.G.); serranr@tcd.ie (R.S.-G.); govanj@tcd.ie (J.G.); 2 St. Petersburg National Research University of Information Technologies, Mechanics and Optics, St. Petersburg 197101, Russia

**Keywords:** magnetic nanoparticles, core-shell, silica, titanium dioxide, photocatalysis, magnetic separation

## Abstract

In this work, CoFe_2_O_4_@SiO_2_@TiO_2_ core-shell magnetic nanostructures have been prepared by coating of cobalt ferrite nanoparticles with the double SiO_2_/TiO_2_ layer using metallorganic precursors. The Transmission Electron Microscopy (TEM), Energy Dispersive X-Ray Analysis (EDX), Vibrational Sample Magnetometer (VSM) measurements and Raman spectroscopy results confirm the presence both of the silica and very thin TiO_2_ layers. The core-shell nanoparticles have been sintered at 600 °C and used as a catalyst in photo-oxidation reactions of methylene blue under UV light. Despite the additional non-magnetic coatings result in a lower value of the magnetic moment, the particles can still easily be retrieved from reaction mixtures by magnetic separation. This retention of magnetism was of particular importance allowing magnetic recovery and re-use of the catalyst.

## 1. Introduction

Core-shell multifunctional magnetic nanoparticles have a wide variety of applications including catalysis, magnetic separation and numerous biomedical uses. There are many approaches of coating of magnetic nanoparticles with various coatings including noble metal, carbon, silica, zirconia, titania and polymer shells [1,2,3,4]. Silica coated-iron magnetic oxide based nanocomposites have attracted particular interest as the silica coating on magnetic nanoparticles gives a number of advantages. It was demonstrated that SiO_2_ coating can protect the nanostructure against degradation and oxidation of the magnetic core (e.g., Fe_3_O_4_ or Fe) [5]. For example, it was shown that a silica coating on γ-Fe_2_O_3_ can prevent the thermal transition of γ-Fe_2_O_3_ to the less magnetic α-Fe_2_O_3_ [6]. As a result the silica-coated magnetic nanoparticles are stable against degradation which improves their biocompatibility and facilitates their utilization. Furthermore, the ease of silica surface modification [7] allows for further specific functionalization to perform catalysis, biolabeling and drug delivery [5,8,9,10,11,12,13,14]. The surface of silica has also been shown to be easily modified with cationic groups which can improve its electrostatic binding to DNA [15] enhancing its use as a drug delivery agent and improving its cellular transport.

Titanium dioxide is another very important material which has also been applied for the coating and functionalization of magnetic nanoparticles [16,17,18]. Titanium dioxide has been used for many years as a white pigment in paint, sunscreen and even toothpaste [19,20]. TiO_2_ nanoparticles have also found important applications in photovoltaic cells (e.g., dye sensitized solar cells), gas sensing, photo-electrodes and as efficient photocatalysts [20,21]. The photo-catalytic trait of TiO_2_ arises from the electron-hole pair that forms upon irradiation of TiO_2_ under UV light. The resultant charge carriers that are formed can then migrate to the surface of the particle where they can react with adsorbed water and oxygen to form oxidative radical species such as peroxide radicals. These radicals can then oxidize organic molecules present at the surface [22]. The ability of TiO_2_ to photo-catalyze the degradation of organic compounds has been exploited in environmental sectors to reduce the levels of organic contaminants in polluted water and polluted air as well as in self-cleaning windows and surfaces [20,23]. TiO_2_ can exist in three crystalline phases, anatase, rutile and brookite. Of these phases the anatase phase displays the highest photo-catalytic activity [24,25]. This phase can normally be produced by heating TiO_2_ particles, between 400 and 600 °C [21].

The main aim of this work is to prepare and investigate cobalt ferrite based nanoparticles which are coated by a double silica/titania shell. It is expected that the introduction of the SiO_2_ coat between the ferrite and the TiO_2_ shell should reduce the recombination processes between the titania shell and ferrite core as the SiO_2_ could act as an insulator against electron donation [26]. We have selected cobalt ferrite as a magnetic core material because it is more thermally robust and stable comparing to magnetic iron oxide and therefore cobalt ferrite should be suitable for the further preparation of multi-shell structures and a potential thermal treatment at high temperatures. Another goal of our work is to test these new CoFe_2_O_4_@SiO_2_@TiO_2_ core-shell magnetic nanostructures as catalysts for the photo-oxidation of methylene blue dye, under UV light and evaluate their potential as magnetically retrievable and re-usable photo-catalysts.

## 2. Results and Discussion

### 2.1. Synthesis and Characterization of CoFe_2_O_4_ Nanoparticles

**Figure 1:**
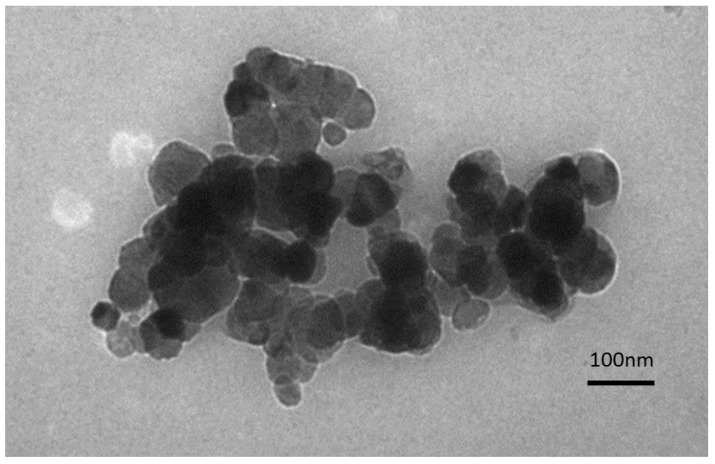
Transmission Electron Microscopy (TEM) image of uncoated CoFe_2_O_4_ nanoparticles.

Cobalt ferrite nanoparticles were synthesized by the co-precipitation of cobalt(II) nitrate and iron(III) chloride in the presence of ammonium hydroxide according to a previously reported procedure [27]. The product was a dark brown/black solid. The magnetic moment of the CoFe_2_O_4_ sample was 85.65 A∙m^2^∙kg^−1^. The Transmission Electron Microscopy (TEM) imaging (Figure 1) have shown the CoFe_2_O_4_ nanoparticles with the size distribution between 40 and 100 nm.

**Figure 2:**
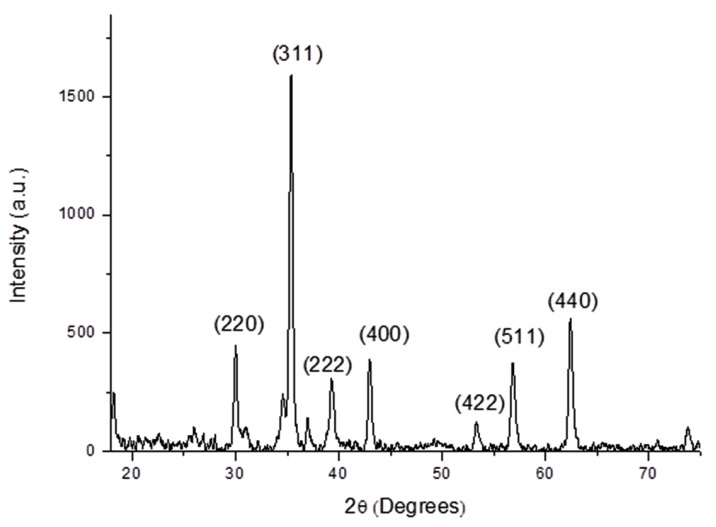
X-ray powder diffraction (XRD) pattern of initial CoFe_2_O_4_ nanoparticles.

The X-ray powder diffraction (XRD) pattern below also confirmed the formation of the CoFe_2_O_4_ phase (Figure 2). The peaks in the diffractogram were in agreement with the expected sample peaks for cobalt ferrite as given in the JCPDS database and relevant literature [28].

### 2.2. Synthesis and Characterization of CoFe_2_O_4_/SiO_2_ Core-Shell Nanostructures

**Figure 3:**
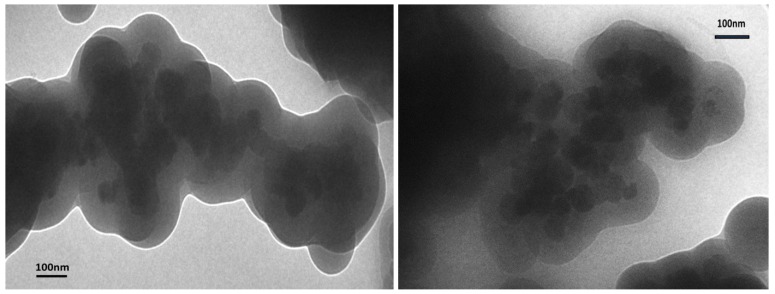
TEM images of the SiO_2_ coated CoFe_2_O_4_ nanoparticles.

Silica coating of the cobalt ferrite nanoparticles was produced using tetraethylorthosilicate (TEOS) and ammonia solution. According to magnetization studies, the magnetic moment of the silica coated cobalt ferrite sample was reduced to 15.99 A∙m^2^∙kg^−1^. This drop in magnetic moment was due to the presence of non-magnetic SiO_2_ coating. This is a significant descent which enables us to suggest that the coating is quite thick. This was confirmed by the TEM data below (Figure 3).

The TEM images (Figure 3) showed a core-shell structure of the nanoparticles, although a large amount of aggregation is observed: the particles sticking together to form clumps and aggregates of particles which are then coated with the SiO_2_. According to TEM, the particles range in size from approximately 40 nm up to 100 nm in the core, with the coatings being approximately 50 nm in thickness. This gives the particles a total size of between 90 and 150 nm. Although these images do not show the ideal structure of a single core contained within a shell, they do confirm the formation of the required core-shell silica-cobalt ferrite nanoparticles.

**Figure 4:**
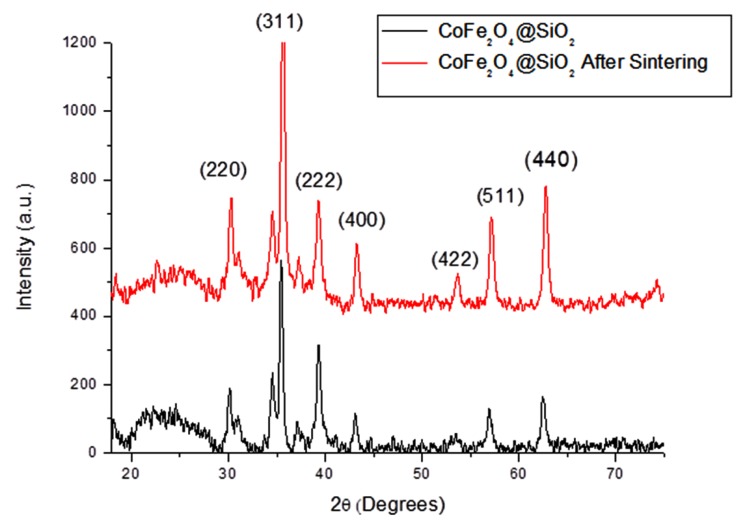
XRD pattern of SiO_2_ coated CoFe_2_O_4_ nanoparticles before and after sintering at 600 °C.

The XRD pattern for SiO_2_ coated CoFe_2_O_4_ nanoparticlesis is shown in Figure 4. Again the dominating peaks present are those of CoFe_2_O_4_ as discussed above. The broad peak at low angles may indicate the presence of the amorphous SiO_2_. By comparison with the JCPDS database for SiO_2_ and the results obtained by Hu *et al.* [29] , the peak at 31 contain a trace of SiO_2_ along with the peak at 2θ ≈ 35, however both of these peaks are highly masked by the CoFe_2_O_4_ peaks in the same area.

### 2.3. Synthesis and Characterization of CoFe_2_O_4_@SiO_2_@TiO_2_ Core-Shell Nanostructures

**Figure 5:**
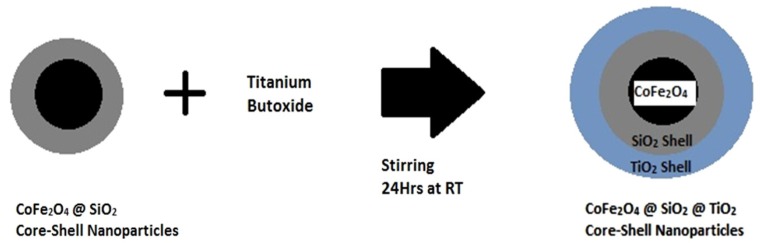
Reaction scheme for the coating of SiO_2_ coated cobalt ferrite nanoparticles with a second TiO_2_ coating.

Titania/silica coated cobalt ferrite nanoparticles have been prepared by hydrolysis of titanium tetrabutoxide precursor in the presence of silica coated cobalt ferrite nanoparticles according to the scheme below (Figure 5).

**Figure 6:**
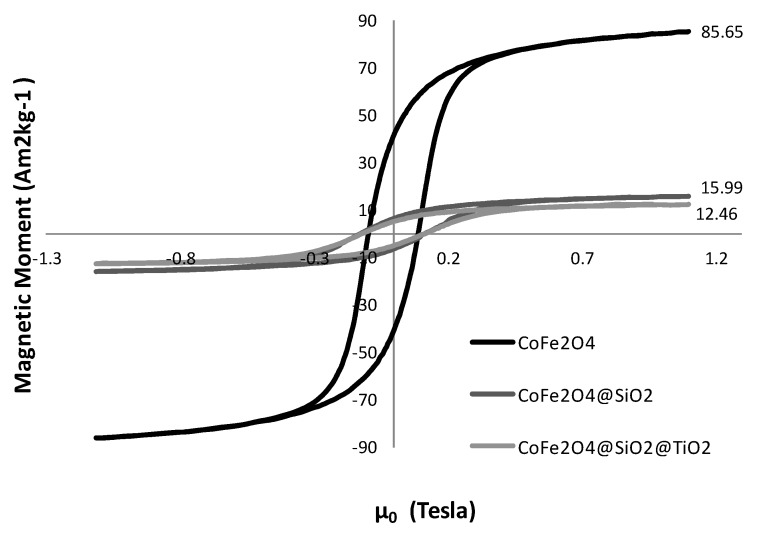
Vibrational Sample Magnetometer (VSM) curves of CoFe_2_O_4_, CoFe_2_O_4_@SiO_2_ and CoFe_2_O_4_@SiO_2_@TiO_2_ coated nanostructures.

After applying the silica/titania double coating, the magnetic moment of the sample dropped again to 12.46 A∙m^2^∙kg^−1^ (Figure 6). This reduction in magnetic moment proves that the size of the non-magnetic coating has increased due to the presence of non-magnetic TiO_2_ shell, resulting in a lower magnetic moment. As the drop in magnetic moment is very low this time, it would indicate that the TiO_2_ coating is very thin in comparison with the SiO_2_ coating. This was also confirmed by TEM results.

**Figure 7:**
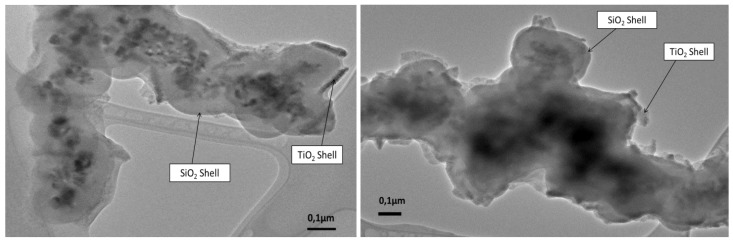
TEM images of the two types of coating around the CoFe_2_O_4_ core, forming the double coated core-shell nanostructures.

TEM images of the double coated sample of CoFe_2_O_4_ are shown in Figure 7. In the TEM images we can clearly see the presence of two types of coating on the CoFe_2_O_4_ nanoparticles. The main coating around the core is a SiO_2_ shell of approximately 50 nm thick. Then the second TiO_2_ coating lies on top of the silica shell. However, the TiO_2_ does not form a complete solid coating on the SiO_2_ but rather some isolated fragments of TiO_2_. Most likely the complete uniform titania coating does not form in this case due to a lattice mismatch between SiO_2_ and TiO_2_.

**Figure 8:**
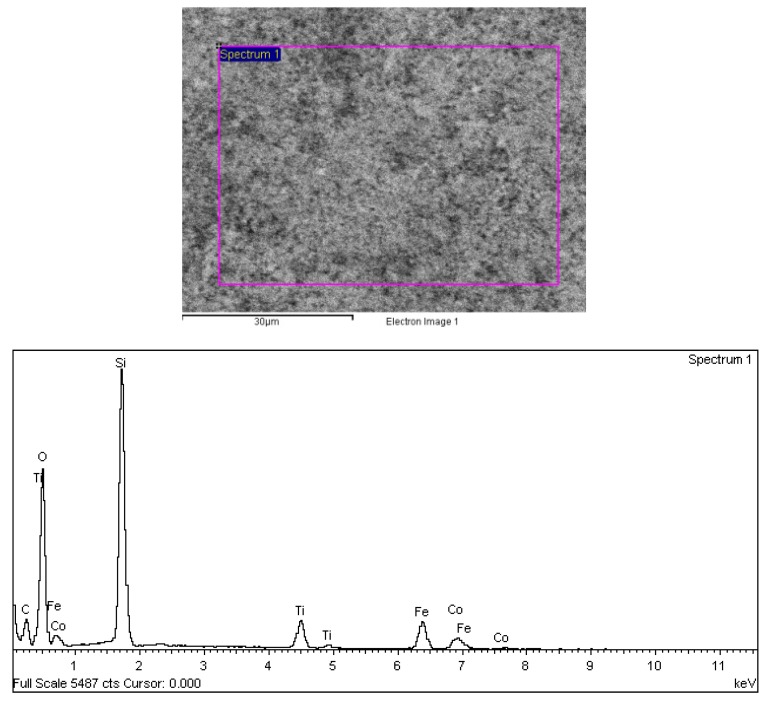
Energy Dispersive X-Ray (EDX) analysis of the TiO_2_/SiO_2_ coated CoFe_2_O_4_ nanostructures.

The Energy Dispersive X-ray (EDX) analysis of the samples (Figure 8) confirms the presence of elements corresponding to CoFe_2_O_4_, SiO_2_ and TiO_2_ further proving the formation of a double coating.

**Figure 9:**
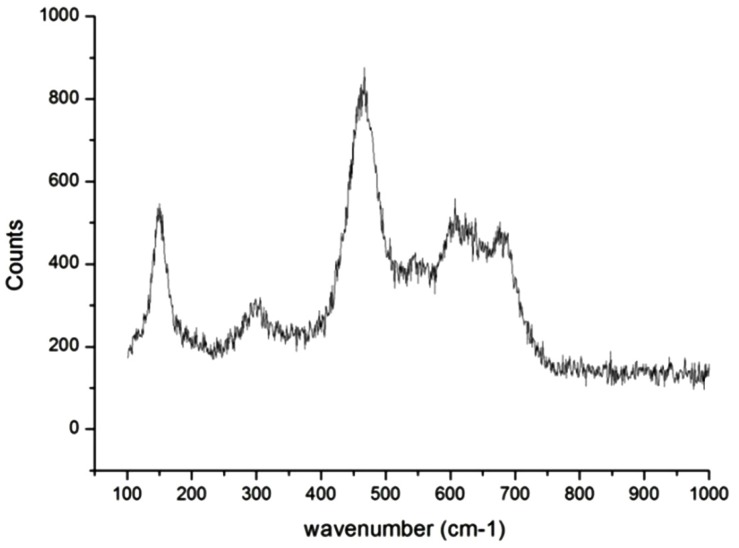
Raman spectrum of CoFe_2_O_4_@SiO_2_@TiO_2_ core-shell nanostructures.

The presence of thermally stable cobalt ferrite core enabled us to perform sintering of the CoFe_2_O_4_@SiO_2_@TiO_2_ core-shell nanostructures at 600 °C in order to produce photocatalytically active anatase and rutile forms of TiO_2_. Figure 9 displays the Raman spectra of TiO_2_**/**SiO_2_ coated CoFe_2_O_4_ nanoparticles which were sintered at 600 °C for 1h. The sharp peak at 146 cm^−1^ is indicative of the presence of TiO_2_ in the anatase phase. Although the rutile phase also exhibits a peak around this area it is a normally very weak signal, therefore this peak is due to the presence of the anatase phase [30]. However the peaks at 450 cm^−1^ and 614 cm^−1^ would suggest the presence of TiO_2_ in the rutile phase [31]. This has most likely arisen due to the heat treatment of the nanoparticles. Some of the TiO_2_ present may have converted from the amorphous phase to the anatase phase while some may have converted further to the rutile phase resulting in a mixture of TiO_2_ phases on the nanoparticle surface. The peaks at 460 cm^−1^ and 680 cm^−1^ indicate the presence of cobalt ferrite. The SiO_2_ peaks are also quite weak and hard to distinguish in this spectrum however the broad peak centered at 300 cm^−1^ in the spectrum indicates the presence of a SiO_2_ shell.

### 2.4. Photocatalytic Activity Testing of CoFe_2_O_4_@SiO_2_@TiO_2_ Core-Shell Nanoparticles

**Figure 10:**
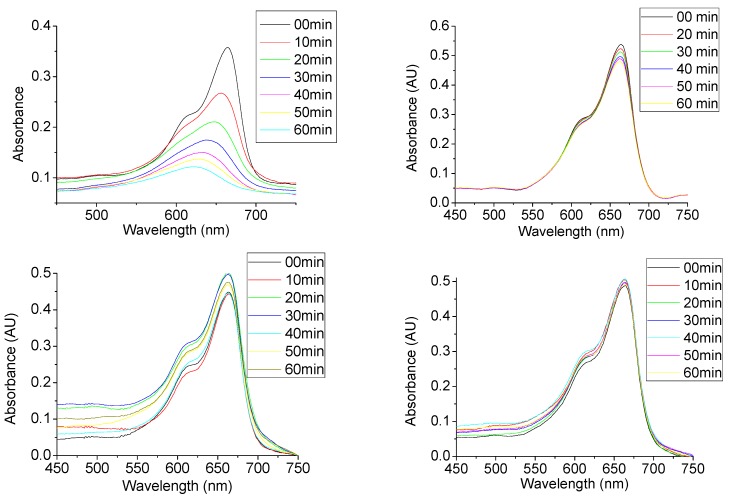
Graphs showing the absorption of methylene blue dye with the catalytic CoFe_2_O_4_@SiO_2_@TiO_2_ nanoparticles present over time (**Top Left**) for catalytic testing and also showing just the dye without the nanoparticles present (**Top Right**), the dye in the presence of CoFe_2_O_4_@SiO_2_ nanoparticles (**Bottom Left**) and in the presence of CoFe_2_O_4_@SiO_2_@TiO_2_ without UV illumination (**Bottom Right**) all as controls.

The CoFe_2_O_4_@SiO_2_@TiO_2_ core-shell nanoparticles sintered at 600 °C were used for photo-catalytic testing. After these nanoparticles were added to the methylene blue solution, the absorbance of the dye reduced much faster as opposed to the control tests. This is a clear indication of photo-catalytic activity of these samples. The Figure 10 (top left) displays the decrease in absorbance of the methylene blue dye over time in the presence of the sintered CoFe_2_O_4_/SiO_2_/TiO_2_ nanoparticles under UV illumination versus that with no nanoparticles present (Figure 10, top right), in the presence of CoFe_2_O_4_@SiO_2_ nanoparticles (bottom left) and CoFe_2_O_4_@SiO_2_@TiO_2_ without UV illumination all as controls (bottom right). As shown, the rate of decrease in absorbance is much higher than that of the control samples. This shows that the TiO_2_ coating is catalyzing the photo-oxidation of the dye molecules as expected. The slight decrease in absorption in the control tests is most likely due to partial bleaching of dye molecules under the UV-light or the absorbance of dye molecules to the surface of the catalyst species in the case of the dark (no UV illumination) control.

**Figure 11:**
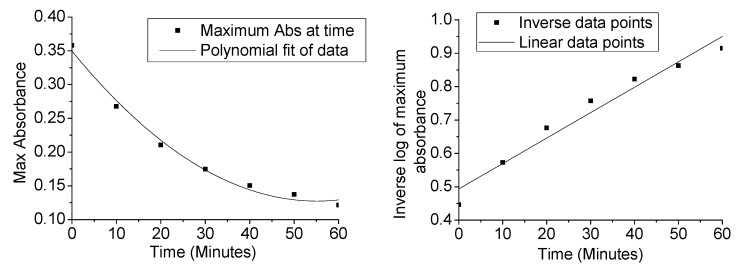
Graph of the changes in the maximum of dye absorbance plotted against time for catalytic CoFe_2_O_4_@SiO_2_@TiO_2_ nanoparticles sintered at 600 °C (**Left**). Graph of Log (1/Max Abs) *versus* time for the catalytic CoFe_2_O_4_@SiO_2_@TiO_2_ nanoparticles sintered at 600 °C (**Right**).

Figure 11 (Left) shows the changes in the maximum of dye absorbance plotted against time for the reaction of the CoFe_2_O_4_@SiO_2_@TiO_2_ catalyst sintered at 600 °C. In the control tests it is observed that the absorbance drops linearly with increasing time. This shows that the bleaching of the dye under UV light is a linear reaction and is independent of the concentration of methylene blue. When the core-shell nanoparticles are added as a catalyst it is clear that we have the first order reaction here according to the plot of the Log (1/Max Abs) versus time (Figure 11, Right). This is in line with previous reports on TiO_2_ nanoparticle based photocatalytic species [18,20,25,32].

**Figure 12:**
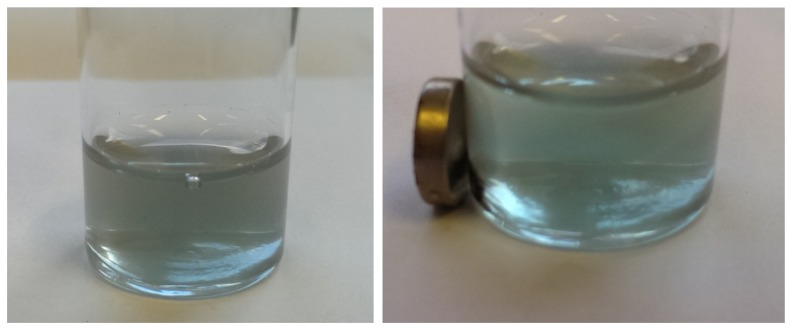
Images of suspended CoFe_2_O_4_@SiO_2_@TiO_2_ in methylene blue solution in water (**Left**) and in the presence of a permanent magnet (**Right**).

Finally, it is very important to notice that the CoFe_2_O_4_@SiO_2_@TiO_2_ nanoparticles of the catalyst were totally recoverable from the reaction mixture using a simple magnetic separation using a permanent magnet as demonstrated in Figure 12.

## 3. Experimental Section

### 3.1. General Procedures

All chemicals were purchased from Sigma-Aldrich and used as supplied unless stated otherwise. Solvents were obtained from the solvent stores at TCD and again were used as received unless stated.

All sonication processing was carried out using either Branson 2510 sonic bath or a Grant Scientific sonic bath operating at 60 Hz. Every magnetic separation was carried out using a 0.5 Tesla magnet.

All TEM imaging and EDX analysis were carried out on a FEI Titan Themis 200 series machine (FEI, Hillsboro, OR, USA).

Raman spectra were recorded using Raman microscope (Renishaw, Gloucestershire, UK) operating at 785 nm using 1% of the 200 mW power.

X-ray diffraction was performed using Siemens-500 X-ray diffractometer (Siemens, Munich, Germany), Cu metal 1.5406 Å. Powder samples were deposited on silica glass using grease to adhere the sample to the glass surface. Overnight patterns were run for all samples. Diffractograms were then compared to the JCPDS database.

Magnetisation measurements were carried out using a custom made permanent magnet variable flux source vibrating sample magnetometer (VSM; home-build, School of Physics, Trinity College Dublin, Dublin, Ireland) at room temperature with field applied up to 1 Tesla.

### 3.2. Preparation of Cobalt Ferrite Nanoparticles in Aqueous Phase

The CoFe_2_O_4_ NPs were prepared based on the method reported by Biswal *et al.* [27] with some modifications. In a 250 mL RBF, cobalt nitrate (Co(NO_3_)_2_**^.^**6H_2_O) (0.6 g/0.002 moles) was dissolved in 100 mL degassed water. To this, iron(II) chloride (0.8 g/0.004 moles) was added followed by the addition of ammonium hydroxide solution, until the cobalt-iron solution measured pH of 11. This mixture was heated in an oil bath to 80 °C and stirred for 1 h. The solution was allowed to cool to room temperature and the product was collected by magnetic separation (1.2 Tesla). The product was washed with Millipore water several times until the pH of the nanoparticle solution was neutral. Then the water was removed by rotary evaporation followed by drying under vacuum using a Schlenk line. The nanoparticulate product was then analyzed using VSM, TEM and Raman techniques.

### 3.3. Preparation of CoFe_2_O_4_@SiO_2_ Core-Shell Nanoparticles

The SiO_2_ coating was performed using the methods reported by Corr *et al.* [5] with some modifications.

In a 250 mL RBF, 25 mL of the citric acid stabilized NP solution (0.028 g of NPs/1.19 × 10^−4^ moles) was mixed with 25 mL ammonium hydroxide in 100 mL of Ethanol. To this solution, TEOS solution (400 μL/0.3732 g/1.791 × 10^−3^ moles) was added. The mixture was sonicated in ice for 1 h. The resultant NPs were extracted again using magnetic separation and were washed several times with EtOH. They were then dried by rotary evaporation. They particles were then analyzed using VSM, TEM, Raman and XRD analysis techniques.

### 3.4. Preparation of CoFe_2_O_4_@SiO_2_/TiO_2_ Core-Shell Nanostructures

The TiO_2_ coating was performed based the method in Pang *et al.* [32] with some modifications. CoFe_2_O_4_@SiO_2_ (0.0495 g) was dispersed by sonication in 100 mL EtOH. Titanium tetrabutoxide (0.4 mL/1.175× 10^−3^ moles) was added by syringe and the solution was sonicated for approximately 5 min. After sonication the mixture was allowed to stir continuously at room temperature for 24 h. The product was then extracted by magnetic separation and washed 3 times with EtOH to clean and the solvent was then removed by rotary evaporation and were analyzed with VSM, Raman, XRD, TEM and EDX analysis to show their composition.

Samples obtained from this procedure were then sintered at 600 °C to study their stability and to produce the catalytically active titania-anatase phase. 

### 3.5. Catalytic Activity Testing of TiO_2_ Coating

Photo-catalytic testing was carried out on the samples containing a TiO_2_ shell based on the methods reported by Pang *el al.* [16]. 10 μL of methylene blue solution (1 × 10^−3^ moles) was added to 3 mL of Millipore water in a quartz cuvette. To this, a few milligrams of nanoparticles were added to the solution and it was allowed to stir in darkness for 1hr. After the hour, a UV-Vis spectrum was recorded for the sample at time *T*_0_. The solution was then placed under a UV light at low wavelength and allowed to stir for 10 min. Another UV-Vis spectrum was then recorded. The sample was then placed back under UV light for another ten minutes and this process was repeated every ten minutes for 1 h. A background of Millipore water was ran before each scan. This entire process was repeated for each sample sintered at different temperatures.

## 4. Conclusions

We have demonstrated that magnetic cobalt ferrite nanoparticles can be prepared and coated by the double SiO_2_/TiO_2_ layer using metallorganic precursors to give new functional “core-shell” nano-structures. Although the SiO_2_ coating is relatively simple to obtain, the complete TiO_2_ coating on silica proves much more challenging to produce, most likely due to the mismatch between the structures of these two materials. The TEM, EDX, VSM and Raman spectroscopy results confirm the presence of the TiO_2_ layer, although the thickness of TiO_2_ coating is very thin. Despite the additional non-magnetic coatings result in a lower value of the magnetic moment, the particles can still be retrieved from the reaction mixture by simple magnetic separation.

We have demonstrated that the sintering of CoFe_2_O_4_@SiO_2_@TiO_2_ at 600 °C allows us to produce photocatalytically active anatase/rutile titania phases at the surface of these nanostructures enabling efficient photocatalytic oxidation of methylene blue under UV light. The retention of magnetism in these core-shell nanostructures is of particular importance allowing the magnetic recovery and re-use of the catalyst.

Further work is however necessary in this field in order to optimize the reaction conditions to improve the thickness and quality both the SiO_2_ and TiO_2_ coatings on the magnetic nanoparticles. Different reaction times may be considered along with different amounts of the TiO_2_ precursors used. Eventually these nanoparticles are expected to be used in photocatalysis and potentially in biomedical applications such as for example antibacterial control systems.
